# Feasibility and usability of a digital health technology system to monitor mobility and assess medication adherence in mild-to-moderate Parkinson's disease

**DOI:** 10.3389/fneur.2023.1111260

**Published:** 2023-03-15

**Authors:** Héloïse Debelle, Emma Packer, Esther Beales, Harry G. B. Bailey, Ríona Mc Ardle, Philip Brown, Heather Hunter, Fabio Ciravegna, Neil Ireson, Jordi Evers, Martijn Niessen, Jian Qing Shi, Alison J. Yarnall, Lynn Rochester, Lisa Alcock, Silvia Del Din

**Affiliations:** ^1^Translational and Clinical Research Institute, Faculty of Medical Sciences, Newcastle University, Newcastle upon Tyne, United Kingdom; ^2^Faculty of Medical Sciences, Newcastle University, Newcastle upon Tyne, United Kingdom; ^3^National Institute for Health and Care Research (NIHR), Newcastle Biomedical Research Centre (BRC), Newcastle University and The Newcastle upon Tyne Hospitals NHS Foundation Trust, Newcastle upon Tyne, United Kingdom; ^4^The Newcastle upon Tyne Hospitals NHS Foundation Trust, Newcastle upon Tyne, United Kingdom; ^5^Department of Computer Science and INSIGNEO Institute for in silico Medicine, The University of Sheffield, Sheffield, United Kingdom; ^6^Dipartimento di Informatica, Università di Torino, Turin, Italy; ^7^McRoberts BV, The Hague, Netherlands; ^8^Department of Statistics and Data Science, Southern University of Science and Technology, Shenzhen, China

**Keywords:** Parkinson's disease, medication adherence, smartwatch, wearable technology, remote monitoring, mobility, inertial measurement units, motor complications

## Abstract

**Introduction:**

Parkinson's disease (PD) is a neurodegenerative disorder which requires complex medication regimens to mitigate motor symptoms. The use of digital health technology systems (DHTSs) to collect mobility and medication data provides an opportunity to objectively quantify the effect of medication on motor performance during day-to-day activities. This insight could inform clinical decision-making, personalise care, and aid self-management. This study investigates the feasibility and usability of a multi-component DHTS to remotely assess self-reported medication adherence and monitor mobility in people with Parkinson's (PwP).

**Methods:**

Thirty participants with PD [Hoehn and Yahr stage I (*n* = 1) and II (*n* = 29)] were recruited for this cross-sectional study. Participants were required to wear, and where appropriate, interact with a DHTS (smartwatch, inertial measurement unit, and smartphone) for seven consecutive days to assess medication adherence and monitor digital mobility outcomes and contextual factors. Participants reported their daily motor complications [motor fluctuations and dyskinesias (i.e., involuntary movements)] in a diary. Following the monitoring period, participants completed a questionnaire to gauge the usability of the DHTS. Feasibility was assessed through the percentage of data collected, and usability through analysis of qualitative questionnaire feedback.

**Results:**

Adherence to each device exceeded 70% and ranged from 73 to 97%. Overall, the DHTS was well tolerated with 17/30 participants giving a score > 75% [average score for these participants = 89%, from 0 (worst) to 100 (best)] for its usability. Usability of the DHTS was significantly associated with age (ρ = −0.560, BCa 95% CI [−0.791, −0.207]). This study identified means to improve usability of the DHTS by addressing technical and design issues of the smartwatch. Feasibility, usability and acceptability were identified as key themes from PwP qualitative feedback on the DHTS.

**Conclusion:**

This study highlighted the feasibility and usability of our integrated DHTS to remotely assess medication adherence and monitor mobility in people with mild-to-moderate Parkinson's disease. Further work is necessary to determine whether this DHTS can be implemented for clinical decision-making to optimise management of PwP.

## Introduction

Parkinson's disease (PD) is a progressive neurodegenerative disorder characterised by cardinal motor symptoms which impact quality of life and independence in individuals with PD; therefore, careful clinical management is of primary importance. Adherence to prescribed medication dosage and timing is vital for effective management of motor symptoms. One of the most effective strategies for managing these symptoms is dopaminergic therapy such as levodopa (1, 2). As PD progresses, increased (3) and/or more frequent (4) doses of levodopa are necessary to ease motor symptoms, but motor complications such as dyskinesias (involuntary movements) and/or motor fluctuations can develop (5). During “ON” periods, symptoms and functional impairment improve following medication intake, whereas “OFF” periods correspond to a worsening of symptoms as the dose wears off (1).

Due to complex medication regimens, adherence is often suboptimal, resulting in poor response to medication, reduced quality of life and increased symptom fluctuation severity (6). It has been shown that over a third of people with PD (PwP; 36.3%, *n* = 45) taking three or more doses of medication daily report poor adherence (6). Modification of complex medication regimens often follows short, infrequent appointments with a clinician, which have been especially affected by the COVID-19 pandemic (7, 8). In addition, patient-clinician interactions are influenced by patient recall and performance bias, and clinicians observe PwP at different stages (“ON,” “OFF” periods) of their medication regimen. Consequently, clinicians often lack adequate insight of daily and habitual motor fluctuations to appropriately adapt medication regimens. This highlights the need for remote and real-world monitoring of mobility and motor symptoms in response to medication in PwP. By objectively modelling and predicting how mobility and motor symptoms change throughout the day in response to medication, clinicians may be able to optimise medication regimens and reduce motor fluctuations in PwP.

Digital health technology systems (DHTSs) have the “potential to transform healthcare research” (9) and present a means for remote monitoring of mobility and assessment of medication adherence and in PwP. Specifically, body-worn sensors [e.g., inertial measurement units (IMUs)] can monitor digital mobility outcomes (DMOs) in an unobtrusive manner, allowing for objective quantification of mobility in PwP (10), such as gait speed (11–13). Other connected devices (e.g., smartphones) provide a valuable indication of contextual factors that affect DMOs (14), such as the likelihood that the individual is indoors or outdoors. Digital health technology (DHT) also presents an avenue to improve individuals' medication adherence in PD, by providing notifications to remind them of their medication intake times (15, 16). A widely used DHT device is the Personal KineticGraph (PKG^®^, Global Kinetics Corp, Australia). The PKG continuously monitors and stores motor symptom data and can send medication reminders (17). However, it does not provide real-time feedback to users, quantify gait components, or register the specific medication taken, therefore limiting its use for comprehensive remote monitoring of PD. Therefore, the first step to enhance customisation and adaptation of medication regimens in PwP, is for research to focus efforts on utilising DHT to comprehensively monitor PwP in their daily life and explore how motor complications and mobility respond to medication. Reducing the burden of complex medication regimens on PwP will improve their quality of life and offer improved management of motor symptoms.

To achieve this, the present study investigates whether a new DHTS integrating a smartwatch, smartphone and IMU can be utilised to monitor mobility and assess medication adherence in PwP. Specifically, the IMU allows for continuous monitoring of DMOs; the smartwatch reminds individuals of their medication intake times and records self-reported intakes through interaction with the digital screen; and the smartphone sends notifications to the smartwatch and records contextual data. Additionally, a diary is filled by participants on a daily basis to record motor complications (i.e., ON and OFF fluctuations and dyskinesia). As highlighted by the World Health Organisation (WHO) (18), feasibility and usability of a DHTS should be amongst the first assessments conducted for the development of new digital health interventions. Indeed, individuals' needs and ability to use DHTS vary with demographic and clinical status, but usability of DHTS is rarely explored (19).

Therefore, as a first step to model how mobility and motor symptoms respond to medication, the present paper aims to investigate: (i) the feasibility and (ii) the usability of the aforementioned DHTS and of a diary to remotely monitor mobility, assess daily medication adherence and track motor complications in PwP. We first hypothesised that the DHTS and motor complications diary will be feasible for PwP, and second, as PD is a progressive disease, that usability of the DHTS's components will decline as participants age and PD progresses. Finally, we provide recommendations and identify potential ways to improve the DHTS for future studies.

## Materials and methods

This section has been prepared following the EVIDENCE (EValuatIng connecteD sENsor teChnologiEs) guidelines for the evaluation of a DHTS in Utility and Usability studies (20).

### Participants and protocol

Participants with PD were recruited as part of the Medical Research Council (MRC) Confidence in Concept (CiC) funded study “Translating digital healthcare to enhance clinical management: evaluating the effect of medication on mobility in people with Parkinson's Disease” (ISRCTN Number: 13156149, https://www.isrctn.com/ISRCTN13156149). This study is also a sub-study of the Mobilise-D—Clinical Validation Study (REC reference: 20/PR/0792) (21).

Due to the paucity of research exploring concurrent real-world mobility and medication adherence in PwP using DHTS, there was insufficient data to inform a reliable power calculation. For this feasibility study, a sample size of 30 was defined according to Consensus-based Standards for the selection of health Measurement Instruments guidelines for measurement properties (22). Ethical approval was obtained from the London—Westminster Research Ethics Committee (REC reference: 21/PR/0469) and the study was conducted in accordance with the declaration of Helsinki (23).

### Eligibility criteria

Eligibility criteria are the same as previously published for the Mobilise-D project (21) and are displayed in Table 1. In the later stages of the disease (Hoehn and Yahr stages IV and V), loss of independence can decrease the ability to perform activities of daily living, this induces difficulties to remotely monitor mobility with IMUs. Additionally, prevalent cognitive impairments associated with disease progression may alter the capacity to utilise the DHTS. Therefore, only people in the early stages of the disease were recruited for this study (inclusion criteria: Hoehn and Yahr stages I to III).

**Table 1:** Inclusion and exclusion criteria for participant recruitment.

**Inclusion criteria**	**Exclusion criteria**
Adults aged 18 or overAbility to consent and comply with any study specific procedures Able to read and write in English Patients with the clinical diagnosis of PD according to the recent criteria of the Movement Disorder Society (24)	Occurrence of any of the following within 3 months prior to informed consent: myocardial infarction, hospitalisation for unstable angina, stroke, coronary artery bypass graft, percutaneous coronary intervention, implantation of a cardiac resynchronization therapy device, active treatment for cancer or other malignant disease, uncontrolled congestive heart disease (NYHA class >3), acute psychosis or major psychiatric disorders or continued substance abuse
Hoehn and Yahr stage I–III On stable Parkinson's disease medication doses (i.e., taking the same medications for 4 weeks or more).	History consistent with Dementia with Lewy Bodies, atypical parkinsonian syndromes (including multiple system atrophy or progressive supranuclear palsy, diagnosed according to accepted criteria)
Able to walk 4 m independently with or without walking aids	Repeated strokes or stepwise progression of symptoms, leading to a diagnosis of “vascular parkinsonism”
Willingness to wear an IMU, a smartwatch and use a smartphone	Drug-induced parkinsonism

### Study protocol

#### Recruitment and screening

Participants were recruited between June 2021 and March 2022 from local movement disorder clinics and from the “Mobilise-D—Clinical Validation Study” at the Newcastle University (UK) site. Potential participants attended an eligibility screening appointment during which the ability to consent was assessed, informed consent was obtained, and eligibility criteria were reviewed.

#### Study assessments

**Figure 1:**
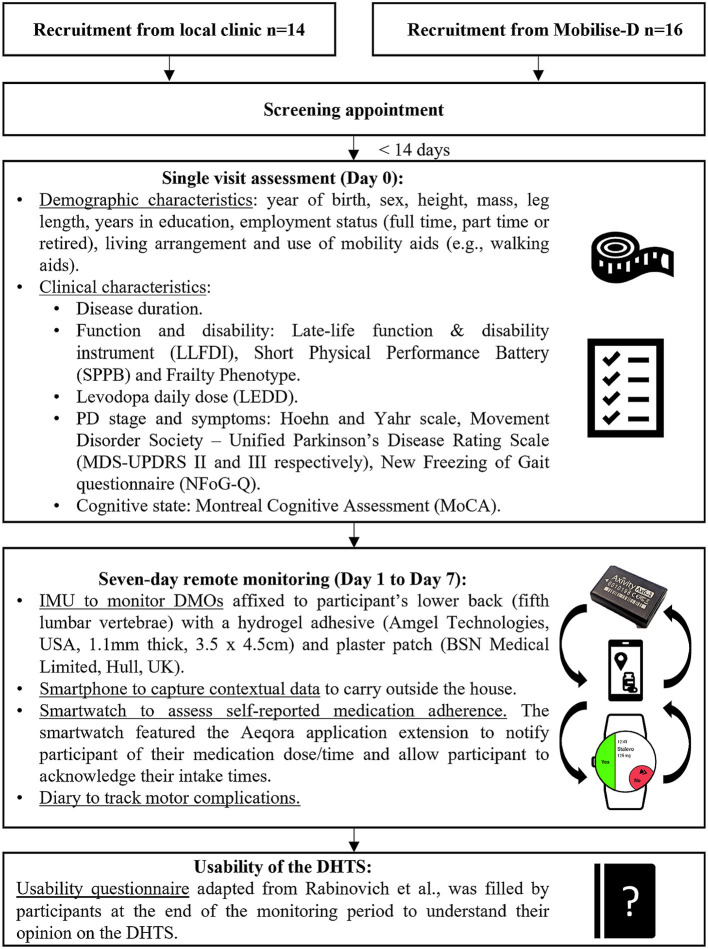
Flowchart of the study protocol.

A flowchart of the study protocol is displayed in Figure 1.

Within 14 days of screening, participants attended a single visit assessment at the Clinical Ageing Research Unit of Newcastle University in which their demographic and clinical characteristics were assessed. Clinical characteristics were measured using validated tools and questionnaires (25–33).

At the end of this visit, participants were equipped with the DHTS and a demonstration of the smartwatch use was made. Detailed written instructions for the day-to-day use of the devices were provided to participants which included the contact details of the research team.

#### Seven-day continuous remote monitoring

The monitoring period started the day after the screening visit, with self-reported medication adherence, mobility and motor complications being monitored over seven consecutive days.

#### IMU to monitor DMOs

To monitor their DMOs, participants wore an IMU [Axivity, AX6, including triaxial accelerometers and gyroscopes, dimensions 23 × 32.5 × 8.9 mm, mass 11 g, frequency 100 Hz, accelerometer range ±8 g, gyroscope range ±2,000 ° degrees per second (dps)] on their lower back (fifth lumbar vertebra) throughout the monitoring period, and were asked to continue their daily activities as usual and not to change their routine.

#### Smartphone to contextualise DMOs

During the monitoring period, participants were also asked to carry a smartphone (Samsung Galaxy S9, S10 or S21, Samsung Group, Suwon-si, South Korea) when leaving their home. The Aeqora mobile application (Department of Computer Science, The University of Sheffield, UK) was pre-installed onto the smartphone to (a) send medication notifications to the smartwatch, and (b) collect contextual information such as weather conditions, geolocation, and the number of steps participants took outside of their home, per day (34). Geolocation data will be used in the future to discern DMOs obtained from indoor and outdoor environments using a deep learning model approach (35).

#### Smartwatch to assess self-reported medication intake

Participants' prescribed medication intake times were sent, *via* the smartphone, to a smartwatch (Ticwatch Pro, Mobvoi) through the custom-made Aeqora application extension, and the smartwatch vibrated to notify participants to take their medication at the programmed intake times. Participants interacted with the smartwatch to acknowledge and log their medication intake times, clicking either “Yes” or “No” on the screen when prompted. Participants were able to input any additional medication intake [Per Required Need (PRN)].

#### Diary to track motor complications

To track motor complications (ON and OFF fluctuations, dyskinesia), participants filled in a paper-based medication diary each day; indicating their “OFF-status” (when participants felt their medication was not working) with an “O” and dyskinesia with a “D.” The diary recorded data over 16-h per day, from 06:00 to 22:00 from Day 1 to Day 7. A copy of the medication diary is provided as Supplementary material 1.

#### Questionnaire to evaluate the usability of the DHTS

At the end of the monitoring period, to evaluate usability of the DHTS, participants completed an adapted version of Rabinovich et al.'s (36) usability questionnaire. The questionnaire used a 5-point ordinal scale, 5 being the most favourable and 1 the least favourable scores, with answers of “no opinion” scored as 3. Participants also provided an overall score for the DHTS, from 0 (worst score) to 100 (best score). Open text questions were added to the questionnaire to allow participants to provide feedback on individual devices and on the DHTS. Specifically, participants were asked to “give any other comments on the DHTS and its devices,” and to describe, where appropriate, the “problems” they had with, and the “features [they] liked” about the DHTS and individual devices. A copy of the usability questionnaire is provided in the Supplementary material 2.

At the end of the 7-day assessment, participants returned all the devices, the usability questionnaire and the motor complications diary through the post using pre-paid tracked envelopes.

### Data processing and analysis

#### Data processing

To gain comprehensive insight into the usability of the DHTS we utilised a mixed methods approach (19). Statistical analysis was carried out using SPSSv28 (IBM, NY). Histograms and boxplots were visually inspected to assess the distribution of the data. Outliers (values that are 1.5 × interquartile range lower or greater than first or third quartiles, respectively) were kept in the analysis. Where appropriate, mean and standard deviation or median and range of the demographic and clinical characteristics were reported.

Qualitative analysis of free text questionnaire responses was carried out by two researchers (EP and HD) who together assessed all participants' responses and developed key themes and subthemes. Individually, the researchers then grouped all responses into these themes and subthemes and finally met to review the groupings and form a consensus.

Data was downloaded from the IMU onto a computer and segmented into seven days and analysed in MATLAB (R2018a, Mathworks, California, United States). Walking bouts (i.e., periods of walking with a minimum threshold of three steps) were identified and gait speed and number of steps per day were calculated from the raw IMU data using validated algorithms in MATLAB (13, 37).

Data logged on the smartwatch and smartphone was uploaded to the secure eScience platform (38) and processed using validated algorithms for the contextual data (34), and manually for self-reported medication intake. Raw data from the smartwatch was exported to .xlsx files and included the following items for each day: medication type, time, dose and participants' input (“Yes” or “No”). The number of hours spent per day, in the “ON-” and “OFF-status” and time spent experiencing dyskinesias, were evaluated using annotated motor complication diaries.

#### Quantitative assessment of feasibility of the DHTS and motor complications diary

The WHO report (18) defines feasibility as “[…] whether the digital health system works as intended in a given context.”

To test whether the DHTS and motor complications diary will be feasible for individuals with PD, we explored the feasibility of the DHTS to measure mobility (IMU) and assess self-reported medication adherence (smartwatch), and the feasibility of the smartphone and diary to collect contextual data and track motor complications (“ON,” “OFF” periods, dyskinesia), respectively. In reference to the WHO definition of feasibility, we assessed whether the intended data had been collected by each device in the system (18).

Concerning medication adherence, the number of interactions expected corresponded to the number of prescribed medication intakes, excluding PRN intakes. As the overall aim of this project is to model mobility and motor complications in response to medication intake and this will include PRN doses, interactions recorded per day included PRN intakes. Duplicates (second intake separated by 30 minutes or less from initial intake) were excluded from the analysis.

Table 2 summarises the measures of feasibility and outcomes extracted.

**Table 2:** Measure of feasibility and outcomes extracted for each device of the DHTS and motor complications diary.

**Device**	**Measure of feasibility**	**Outcomes extracted**
IMU	Percentage of IMU datasets collected over 7 days	Gait speed and number of steps per day.
Smartphone	Percentage of datasets collected over 7 days and percentage of days missing.	Number of steps taken outside the home per day.
Smartwatch	Percentage of participants interacting with the smartwatch over 7 days.	Number of interactions recorded.
Motor complications diary	Percentage of diaries returned and legible.	Time spent in ON or OFF state and dyskinesia.

#### Quantitative assessment of usability of the DHTS

The WHO report (18) defines usability as “[…] whether the digital health system can be used as intended by users.”

The usability of the DHTS was evaluated through analysis of the quantitative part of the usability questionnaire. To test our second hypothesis that usability of the DHTS's devices will be affected by participants' demographic and clinical characteristics, we ran Spearman's rho correlations between the overall usability score of the DHTS provided by participants and their demographic and clinical characteristics. Considering the lack of normality of the sample's DHTS usability score, we used bootstrapping correlations with bias corrected and accelerated 95% confidence interval (BCa 95% CI) to improve the accuracy of the confidence interval [for information on bootstrapping, please see Wright, London and Field's paper (39)]. It was anticipated that usability of the DHTS would decrease with age or disease progression. Therefore, correlation analysis was run between the overall DHTS score (0–100) and demographic (age) (α = 0.05), as well as clinical characteristics (disease duration, number of medication doses prescribed per day, SPPB score, MDS-UPDRS II and MDS-UPDRS III scores, frailty phenotype and total NFoG-Q score) characteristics. Concerning the correlation between the overall DHTS score and participants' clinical characteristics, because we would reject the null hypothesis should any of the seven clinical characteristics be correlated with the overall DHTS score, a Bonferroni correction was performed and α adjusted to 0.007.

#### Qualitative assessment of feasibility and usability of the DHTS

To identify opportunities to improve the DHTS, we evaluated the qualitative part of the usability questionnaire (19, 40, 41). To analyse these responses, we took a hybrid approach using both deductive and inductive methods, originally grouping qualitative feedback into feasibility, usability, and recommendations for improvement. From exploration of responses, acceptability was included as an additional theme and, based on a previous definition (42), refers to the extent to which the DHTS is perceived as agreeable. Finally, the quantitative part of the usability questionnaires was analysed again with questions grouped according to the identified theme. Question 3 was the only question relating to the feasibility theme. Questions 1, 2, 7, and 8 related to the usability theme and the remaining questions (4, 5, 6, 9, 10, 11, and 12) related to the acceptability theme.

## Results

### Demographic and clinical characteristics

Thirty participants (22 males, 63 ± 9 years, levodopa equivalent daily dose 676 ± 370 mg·day^−1^) who met the inclusion criteria (Table 1) were included in this study. Most (*n* = 29) participants were at Hoehn and Yahr stage II (97%), and one was at Stage I (3%). Participants' clinical and demographic characteristics are presented in Table 3.

**Table 3:** Demographic and clinical characteristics of participants recruited for the study.

**Characteristics**	**Mean ±SD**	**Median (Min–Max)**	**Frequency**
Males/females			22/8
Age (years)	63 ± 9		
BMI (kg·m^−2^)	26.2 ± 4.2		
Education (years)	13 ± 3		
Disease duration (years)		5 (1–17)	
N° doses prescribed per day		5 (3–13)	
Hoehn and Yahr stage, stage: *n* (%)			I: 1 (3%)II: 29 (97%)
LLFDI function (0–160)	132 ± 19		
LLFDI function walking device (0–40)		33 (32–35)	
LLFDI disability frequency (0–80)		56 (43–70)	
LLFDI disability limitation (0–80)	66 ± 9		
LEDD (mg·day^−1^)	676 ± 370		
Frailty Phenotype, phenotype: *n* (%)			0: 16 (53%) I: 10 (33%) II: 3 (10%) III: 1 (3%)
MDS-UPDRS Part II (0–52)		11 (2–33)	
MDS-UPDRS Part III (0–132)		30 (7–43)	
NFoG-Q (0–33)		0 (0–26)	Score ≥ 1: *n* = 10
MoCA (0–30)		28 (21–30)	
SPPB (0–12)	10 ± 1		

Outcomes are reported as mean and standard deviation (SD) when normally distributed and median with minimum and maximum values (Min–Max) when they lack normality.

BMI, body mass index; LLFDI, late-life function and disability instrument; MDS-UPDRS, movement disorder society-unified Parkinson's disease rating scale; NFoG-Q, new freezing of gait questionnaire; MoCA, Montreal Cognitive Assessment; SPPB, short physical performance battery test.

No serious adverse event was reported.

### Quantitative assessment of the feasibility of the DHTS and motor complications diary

#### IMU to monitor DMOs

IMU data was collected for 93% of participants (*n* = 28) over the 7-day monitoring period. Two data sets were missing because one DHTS was recalled due to technical issues with the smartwatch and one participant removed it on day 3 due to skin irritation. Averaged over the 7 days monitored, the median gait speed collected from the IMUs was 1.04 m·s^−1^ and ranged from 0.90 to 1.28 m·s^−1^. The median number of steps (indoor and outdoor) recorded per day by the IMU ranged from 11,228 to 13,693.

Table 4 shows participants' gait speed and number of steps per day recorded by the IMU from day 1 to day 7.

**Table 4:** Gait speed and number of steps per day measured from the IMU's data (*n* = 28).

	**Gait speed m·s^−1^**	**Number of steps per day (inside and outside)**
	**Median (Min–Max)**	**Median (Min–Max)**
Day 1	1.05 (0.76–1.16)	13,235 (3,688–24,556)
Day 2	1.05 (0.76–1.18)	13,092 (2,935–24,099)
Day 3	1.04 (0.83–1.16)	13,693 (4,360–38,655)
Day 4	1.03 (0.75–1.20)	11,919 (2,387–29,719)
Day 5	1.05 (0.82–1.14)	11,228 (917–33,403)
Day 6	1.05 (0.85–1.17)	13,399 (1,896–30,872)
Day 7	1.06 (0.85–1.40)	11,823 (1,755–27,394)

#### Smartphone to contextualise DMOs

Contextual data for one participant was missing for the whole monitoring period (Day 1–7), therefore contextual data was recorded for 97% (*n* = 29) of participants. No data was recorded (phone off) for eight participants for 1–5 days [total number of days without contextual data = 23 (11%)]. The median number of steps (outdoor) recorded per day by the smartphone ranged from 313 to 3,307.

Table 5 shows participants' number of steps outside their home environment recorded by the smartphone from day 1 to day 7 (different from the IMU that continuously collected steps per day indoors and outdoors).

**Table 5:** Number of steps taken outside the home, per day, recorded by the smartphone (*n* = 29).

	**Median (Min–Max)**
Day 1	3,307 (0**–**12,068)
Day 2	2,218 (0**–**15,932)
Day 3	1,437 (0**–**19,184)
Day 4	1,521 (0**–**9,014)
Day 5	313 (0**–**11,915)
Day 6	2,425 (0**–**10,342)
Day 7	1,873 (0–19,452)

#### Smartwatch to assess self-reported medication intake

Three participants (10%) did not interact with their smartwatch during the monitoring period, whilst the other 27 participants (90%) interacted with it at least once. Due to technical issues, eight participants (27%) stopped using the smartwatch during the monitoring period, therefore only 22 participants (73%) had smartwatch data recorded over the monitoring period. Delayed (“No” interaction followed by late additional interaction to report intake) and PRN intakes mean that some participants interacted with their smartwatch more than expected. Ninety expected interactions were missing from the records and 191 duplicates were excluded from analysis.

**Figure 2:**
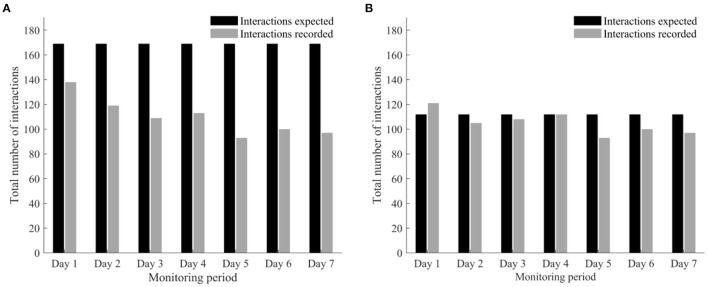
Bar chart representing the number of interactions with the smartwatch expected (black) vs. recorded (grey) per day for all 30 participants **(A)** and only the 22 participants who recorded medication intake for seven days **(B)**.

Figure 2 shows the number of interactions recorded vs. expected per day for (A) the whole sample (*n* = 30), and (B) participants (*n* = 22) who used the smartwatch throughout the monitoring period.

#### Diary to track motor complications

Twenty-nine participants (97%) returned their motor complication diaries, among these, two diaries could not be analysed (not legible) and were excluded from analysis; therefore data from 27 participants (90%) was analysed. Participants spent most of their time in the “ON” state during the monitoring period, with participants' median time in the “ON” state being 108 h, ranging from 56.5 to 112 h. Participants' median time in the “OFF” state was 2 h, ranging from 0 to 55.5 h, over the seven days monitored 19 out of 27 participants reported “OFF” periods. Participants' median time over which they reported dyskinesia was 0 h and ranged from 0 to 30.5 h, 10 out of 27 participants reported dyskinesia over the monitoring period.

Table 6 displays the time spent in each state from Day 1 to Day 7 in 27 participants for whom diary data could be analysed.

**Table 6:** Time (hours) in each medication state (ON, OFF, dyskinesia) recorded from the medication diary (*n* = 27).

	**ON**	**OFF**	**Dyskinesia**
Day 1	15.3 (8.5–16.0)	0.5 (0.0–7.0)	0.0 (0.0–3.5)
Day 2	15.5 (8.5–16.0)	0.5 (0.0–7.5)	0.0 (0.0–4.0)
Day 3	15.5 (6.5–16.0)	0.3 (0.0–9.5)	0.0 (0.0–5.0)
Day 4	15.5 (8.0–16.0)	0.0 (0.0–8.0)	0.0 (0.0–4.5)
Day 5	15.5 (6.5–16.0)	0.0 (0.0–9.5)	0.0 (0.0–5.5)
Day 6	15.5 (8.5–16.0)	0.5 (0.0–7.5)	0.0 (0.0–3.5)
Day 7	15.8 (8.5–16.0)	0.3 (0.0–6.5)	0.0 (0.0–4.5)

### Quantitative assessment of usability of the DHTS

Twenty-eight participants (93%) returned their usability questionnaires. Briefly, 82% of those who returned their questionnaires had little to no trouble getting started with the DHTS (Q1), 64% found the system easy to put on and take off (Q2), and 59% reported experiencing technical issues (Q3). Additionally, the DHTS did not interfere with normal activities in 89% of participants (Q4), with 93% of them felt comfortable wearing the DHTS (Q5), none of the participants felt embarrassed wearing the smartwatch (Q6), and over 68% of participants found that the instructions were clear and that the daily use of the DHTS was easy (Q7 and Q8). According to 75% of participants the system was not bulky or heavy (Q9). Eight percent of them felt that the DHTS bothered them in bed (Q10), and 7% of participants felt that their privacy was invaded by the DHTS (Q11). Finally, 43% of participants reported that they would be happy to wear the DHTS for over a week if their doctor asked them to, 43% of them reported that they would be happy to wear it for a week and the remaining ones less than a week (Q12).

**Figure 3:**
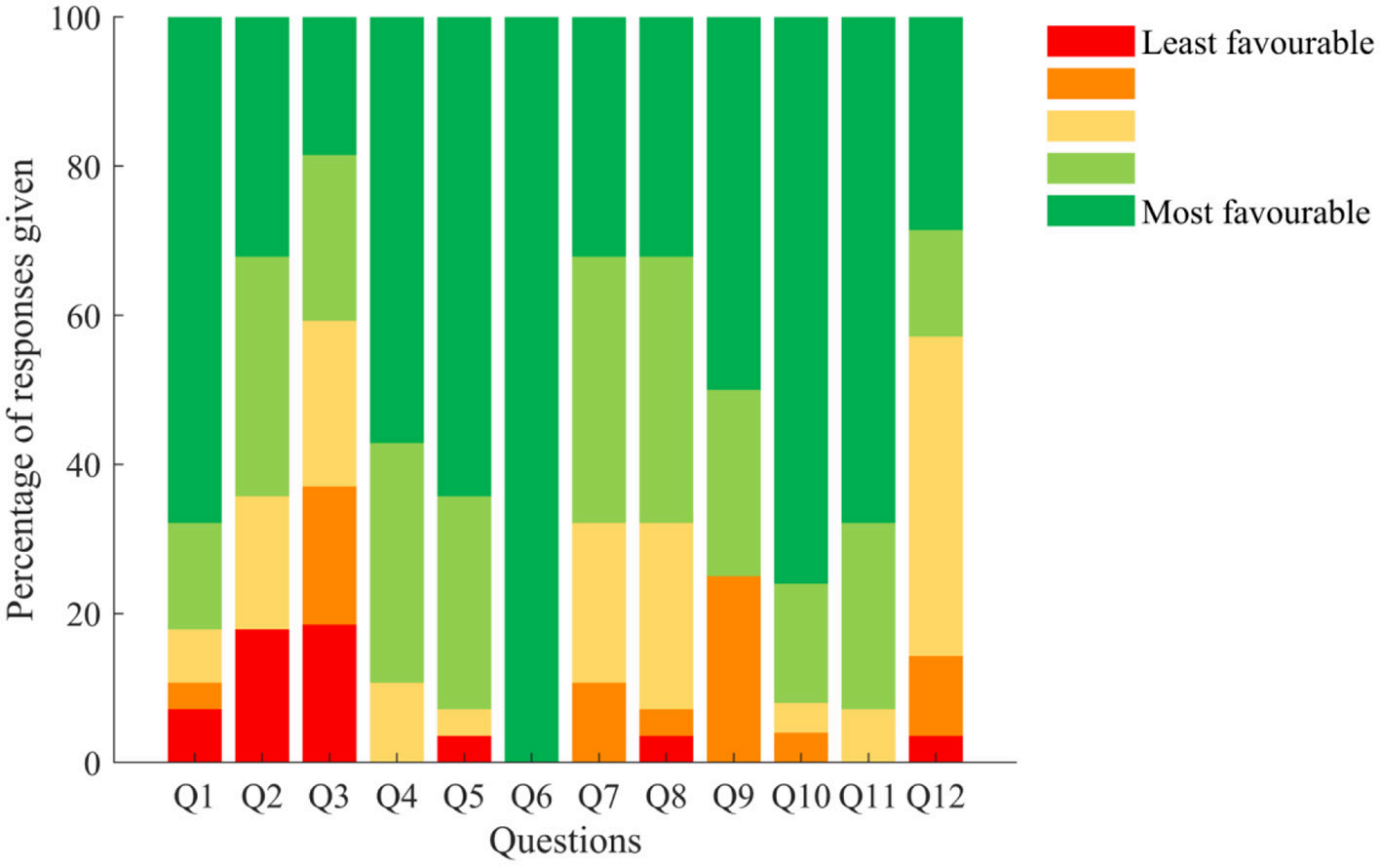
Responses given to the usability questionnaire (%). Q1: How much trouble did you have getting started with the wearable technology system? Q2: The wearable technology system was easy to put on/take off. Q3: I experienced technical problems with the wearable technology system. Q4: The wearable technology system interfered with my normal activities. Q5: I felt comfortable wearing the wearable technology system. Q6: I felt embarrassed wearing the wearable technology system. Q7: The instructions on how to use the wearable technology system were clear. Q8: Using the wearable technology system on a daily basis was easy. Q9: The wearable technology system was bulky/heavy. Q10: The wearable technology system bothered me in bed. Q11: I felt my privacy was invaded by the wearable technology system. Q12: If my doctor would like to use the wearable technology system to assess my activity and medication adherence I would be willing to wear it and use it for. Colour code from red (score = 1 for least favourable response) to green (score = 5 for most favourable response). For this questionnaire, the term “wearable technology system” refers to the DHTS.

Results of the usability questionnaire are presented in Figure 3.

**Figure 4:**
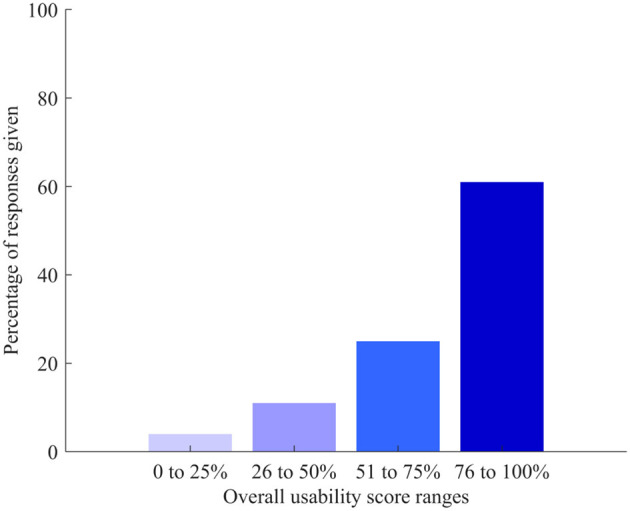
Ranges of overall score given to usability of the DHTS (from 0 worse to 100 best score).

The median overall usability score given to the DHTS was 80% and ranged from 10 to 100%, on a scale ranging from 0 (worst) to 100% (best score). Responses were ranked over 25% intervals, which showed that 61% of participants (*n* = 17) provided scores in the highest rank (score above 75%) and overall, 86% of participants (*n* = 24) found the DHTS usable (score 50% and above) (Figure 4).

**Figure 5:**
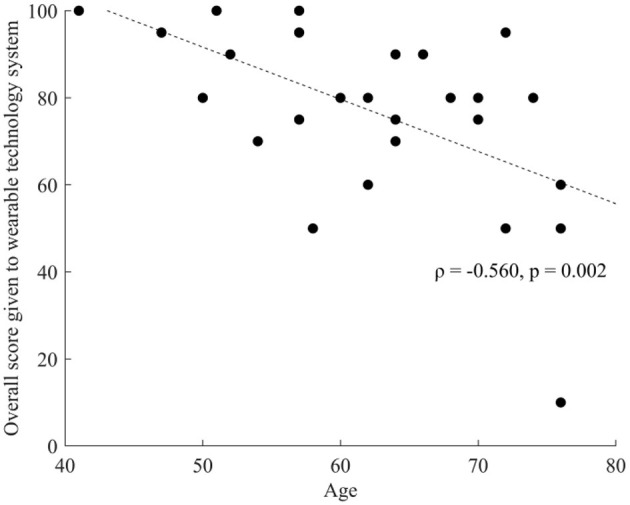
Spearman's rho correlation between overall usability score and age.

A significant correlation was found between the overall usability score and participants' age (ρ = −0.560, *p* = 0.002, BCa 95% CI [−0.791, −0.207]). A scatter plot of the significant correlation is presented in Figure 5; Table 7 shows all the correlation results.

**Table 7:** Correlations between the overall usability score provided by participants on the DHTS in the usability questionnaire and demographic and clinical characteristics.

**Correlation with overall usability score**	**Spearman's rho**	**Sig. (*p*)**	**α**	**BCa 95%CI**	
				**Lower boundary**	**Upper boundary**
Age	**−0.560**	**0.002**	**<0.05**	**−0.791**	**−0.207**
Disease duration	−0.269	0.167	**<**0.007	−0.643	0.145
Number of medication doses per day	−0.309	0.109	**<**0.007	−0.680	0.153
SPPB score	0.412	0.029	**<**0.007	0.018	0.712
MDS-UPDRS II	−0.218	0.266	**<**0.007	−0.536	0.193
MDS-UPDRS III	0.093	0.638	**<**0.007	−0.285	0.500
Frailty phenotype	−0.224	0.251	**<**0.007	−0.582	0.142
NFoG-Q	−0.259	0.184	**<**0.007	−0.581	0.144

Bold values are significant.

### Qualitative assessment of feasibility and usability of the DHTS

Three themes (feasibility, usability, and acceptability) were identified from analysis of participants' feedback of the DHTS and individual devices (open text questions of the usability questionnaire). Responses to the questionnaire (classified into themes and subthemes) are presented in the Supplementary material 3.

#### Feasibility

Feasibility comments were split into two sub-themes: technical and non-technical. Overall, most comments referred to technical issues with the smartwatch, especially concerning its expected function, with some participants reporting that notifications were not delivered at the correct time “*notifications sometimes late*.” Two participants felt the notification vibrations were not strong enough to be felt “*couldn't feel the vibration*.” Non-technical comments encompassed all the devices and reflected the overall satisfaction of participants. Commonly reported comments indicating no issues with feasibility included “*No problems*” and “*All good*.”

#### Usability

Usability was split into three sub-themes: ease of use, disease specific comments and requirement for external support. Usability comments generally reflected issues experienced with the IMU and smartwatch.

Ease of use comments generally concerned the IMU and smartwatch. Regarding the IMU, participants commented on whether they had to replace the attachment during the monitoring period. Most participants who commented on the usability of the IMU were satisfied by the product “*No maintenance! Okay in shower*,” “*Easy to wear*,” but one reported having to “*reapply twice during the 7 days*.” Concerning the smartwatch, two participants found it easy to use, but three expressed their concern about how it is “*easy to get confused*” or the watch being “*over complicated*.” Another participant provided mixed feedback, stating that it was “*Difficult to fasten and unfasten* […] *Clear readable face. Easy to recharge*.”

Disease specific comments related to the participants' tremors adversely influencing their capacity to interact with the smartwatch “*screen is quite small, especially hard with a tremor*” and to reach the IMU (i.e., on their lower back) “*needed help reapplying after showers as could not reach*.” Linking with this, four participants required, or expressed the need for external support to reattach individual devices, “*tricky to attach without help*.”

#### Acceptability

Acceptability was split into four sub-themes: appearance, perception of the device, routine and wearability. Overall, the acceptability comments on the DHTS were positive, with the devices described as “*great to monitor people*” and “*ideal for use*.”

Comments on appearance solely concerned the smartwatch and smartphone. Participants reported that the smartwatch was “*bulky*” or “*too large*” for their wrist. Two participants reported that the watch was “*nice looking*” and had a “*clear screen*.” Concerning the smartphone, participants also found it “*a bit bulky*” and “*too big*” to be carried in a handbag or pocket. One participant commented that the DHTS “*would be a very helpful piece of technology if watch was smaller*.”

Concerning participants' perception of the devices, they liked that the IMU was “*waterproof* .” The smartwatch was reported as “*handy*” and participants liked having a “*reminder to take medication*,” one participant reported having invested in their own smartwatch as a result of the study: “*Seemed like a good idea as I regularly need an alarm reminder. I like the theory. Have invested in my own vibrating alarm watch*” and one participant felt that the notifications helped them realise that their medication “*ran out sooner*” “*No problems just made me realise my meds ran out sooner before next dose*.” Concerning the smartphone, one participant reported that they “*Didn't feel any benefit from this device. A nuisance*.”

Relative to the sub-theme of routine, many participants felt the IMU was “*small*” enough that it could be forgotten about. One participant reported no issues sleeping with the IMU device on “*No problem sleeping. Kept it on all week*.” One participant reported having reservations before wearing it, but quickly forgot this once attached “*Had reservations before wearing it. However, after it was fitted, I soon forgot about it, and it was no trouble*.” One participant felt that “*When working full-time it was annoying having to keep setting the watch*.” Concerning the smartphone, two participants reported either forgetting to take their smartphone with them “*Ok I forgot to take it twice*” or that “*Having to remember to have it with me all the time was annoying*.” In contrast, two participants reported that they were not concerned by having the smartphone with them “*I did not need to do anything with the phone other than have it with me all the time*,” “*Most people carry a phone these days, so no problem for me*.” Participants felt that overall, the DHTS “*didn't interfere with daily life too much*” and that it was “*quite easy to live with*,” with “*little impact on day-to-day activities*.”

Concerning wearability, many participants felt that the IMU was “*comfortable*” and “*unobtrusive*” although some reported occasional itching and skin irritation due to the adhesive. One participant reported that the smartwatch was the “*least comfortable*” device, and one that it was “*a bit big for me*,” but two participants reported being overall satisfied “*Ok to wear*,” “*Not heavy*” by the smartwatch. Three participants reported that the smartphone was “*too heavy*” or “*on the heavy side*.” One participant reported that the overall system was “*Wearable for a week*” with another reporting that they had “*no problems wearing it*.”

**Figure 6:**
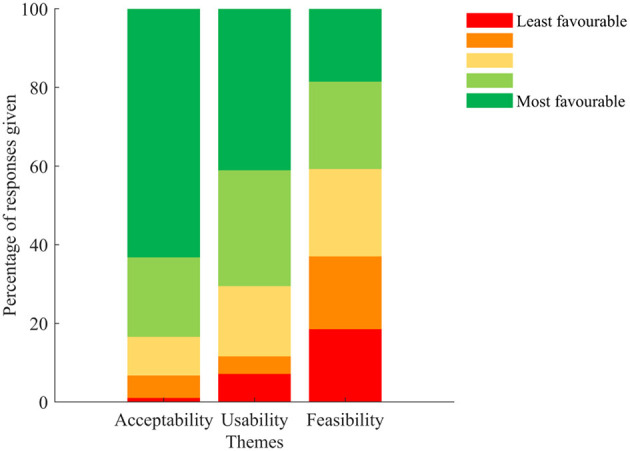
Percentage of responses given to the usability questionnaire classified according to the acceptability (Q4, Q5. Q6, Q9, Q10, Q11, and Q12), usability (Q1, Q2, Q7, and Q8), and feasibility (Q3) themes. Colour code from red (least favourable) to green (most favourable).

Overall, the DHTS was well accepted and usable, but technical issues with the smartwatch affected participants' opinion on the system. Re-analysis on the questionnaire and grouping of each question within the feasibility, usability and acceptability themes, showed that the DHTS was deemed acceptable and usable with 83 and 71% of participants responding with the 2 most favourable answers, respectively. Reflecting the responses given to Q3, only 41% of participants responded with the 2 most favourable answers to the feasibility question. Figure 6 shows the percentage of responses given for each theme.

## Discussion

This study aimed to provide evidence that a new multicomponent DHTS and a motor complications diary could monitor mobility and contextual factors, assess self-reported medication adherence and track motor complications in people with mild-moderate Parkinson's disease, and to identify potential means to improve the DHTS for future use. Results showed that the DHTS is both feasible and usable for remote monitoring of PwP, but the smartwatch was prone to technical issues making it the least feasible and usable component.

### Feasibility

For this feasibility study, the performance of the DHTS was not directly compared to other systems. However, previous research assessing the feasibility of a DHTS in PwP found a completion rate between 62 and 68% over 6 and 13 weeks, respectively, in people mostly scoring II on the Hoehn & Yahr scale (43). Therefore, as previously done by others over longer time periods (12 weeks) in people with a median Hoehn & Yahr score of II, a completion rate of 68% was used as feasibility threshold in the present study (44). Here, this threshold was exceeded for all devices which was expected as the monitoring period was much shorter. Specifically, 97 and 93% of the smartphone and IMU data was collected, respectively, 90% of the motor complications diaries were legible, and 73% of participants reported medication intakes over the 7-day monitoring period. This last completion rate, although lower than that of the smartphone and IMU, remains in line or slightly higher than the compliance to a DHTS use measured over the first week by other authors (about 75% on Day 1 and down to about 65% after few days) (43). Therefore, this study showed that it is feasible to assess self-reported medication adherence and monitor mobility in PwP using this DHTS, and to track motor complications with the diary. However, participants experienced technical issues existed, particularly with the smartwatch, which reduced the feasibility of the DHTS to assess self-reported medication adherence. Importantly, some participants reported not receiving or feeling the smartwatch vibrations, or notifications being late. At this stage, it is difficult to evaluate whether these failures were due: to participants not noticing the vibration, possibly due to a disease related higher threshold to vibrations of the sensory system (45); to the vibration not being strong enough; or to technical failures, leading to notifications not being sent to and/or received by the smartwatch. To appropriately control motor symptoms and complications in PD, strict adherence to prescribed medication timing is crucial. Therefore, the late delivery of smartwatch notifications is a prominent issue that will need to be addressed before this smartwatch can be utilised in future work monitoring medication adherence in PD.

Contextual data (obtained from the smartphone) were missing for 23 days in total (11% of total number of days monitored). Out of these 23 days with missing contextual data, 21 days correspond to participants (*n* = 6) who stopped interacting with their smartwatch during the monitoring period. This may be due to participants forgetting to take the phone with them when leaving their home, and/or them not being aware of the importance of taking the phone with them when they leave their home. As contextual data was collected when the smartphone was on and movement was detected, even when they did not leave their home, missing data for the remaining 2 participants (1 day missing for each participant) may mean that the smartphone was turned off, either voluntarily or because it had run out of battery, and/or that the phone was not moved that day. This may be due to participants not endorsing the purpose or benefit of the device: exemplified in the questionnaire response “*Didn't feel any benefit from this device. A nuisance*.” This is supported by previous research on DHTS observing that older adults and PwP better adhere to device use when they understand their benefits (46, 47). Therefore, to improve participants' adherence to smartphone usage, research should emphasise to participants the importance and purpose of collecting contextual data using a smartphone when monitoring PD symptoms. As technology progresses and sensors reduce in size, we expect the smartwatch to collect contextual data independently and therefore the smartphone to become redundant, which would resolve this issue.

Finally, two diaries were not legible. One because the participant coloured the slots of the diary instead of differentiating between OFF-status or dyskinesia with an O or D, and one because the “O”s and “D”s were not distinguishable. Future work should utilise devices which distinguish between these medication phases. Although this is possible with the PKG (17), as previously stated, it is limited in other outcomes it can produce. Therefore, an optimal device should independently monitor motor symptoms and complications, mobility, contextual factors and self-reported medication adherence.

### Usability

Overall, participants in this study considered the DHTS usable, however the usability score given by participants was negatively correlated with age, indicating that younger adults felt more at ease using the DHTS. This may be a consequence of the lack of experience with DHT associated with advanced age (48), or a lack of confidence associated with handling new technology observed in older adults with PD (49). This suggests that participants may have benefitted from more practice time. No correlation was found between the overall usability score of the DHTS and clinical characteristics of participants. This is surprising since two participants reported having issues interacting with the IMU or smartwatch due to their tremor (see Supplementary material). These results may suggest that a larger and more diverse sample will be necessary to understand the usability of this DHTS in participants with more severe PD, impaired motor function and dexterity issues. Therefore, any attempt to assess self-reported medication adherence in participants with more advanced PD using this DHTS should be preceded by an appropriate usability study.

### Acceptability

Previous research highlighted that for a DHTS to be acceptable, it should, among others, be easy to wear and be aesthetically pleasing (9). Concerning wearability, many participants commented on the “bulky” nature of the smartwatch. We received mixed feedback about the devices, particularly the smartwatch was considered “*too big*” and “*bulky*” by some participants but too small to use with a tremor by others. These findings follow previous research that suggests that wearable devices should be adjusted to individuals' needs and motor symptoms (49). Participants should therefore be offered a range of models between which they are free to choose based on personal preference. This would require careful study design to avoid variations in response due only to a specific choice of DHTS, but may encourage greater compliance, given active participation in model selection.

Forty-three percent of participants would be happy to wear the DHTS for a week and another 43% would be happy to wear it for over a week. This is lower than previously reported (36). In the present study, this was explored for the DHTS as a whole whereas it was investigated for individual devices in Rabinovich et al.'s study. Hence, the acceptability of the DHTS here was probably lowered by the issues experimented with the smartwatch. The COVID-19 pandemic highlighted the need for long-term remote monitoring of people with chronic disease, such as PD, therefore an independent feasibility study of longer duration would be required to apply these results for clinical management of PwP. Previous work (50) observed that the majority of participants with PD would not feel at ease wearing sensors, such as the Axivity sensor in public on visible body locations. Although our study did not specifically ask about wearing the devices in public, wearing a device for a week or longer would most probably involve wearing it in public, as participants in our study did. The greater acceptance of using our DHTS may result from our devices being small, and easily hidden by clothing. Additionally, although many participants in our study were willing to utilise the DHTS, previous studies have highlighted that they do not want this as a replacement for clinical consultations with participants often prioritising communication with their clinician (47, 49).

Despite utilising medical grade adhesive to secure the IMU, one participant stopped the trial due to skin irritations and three others reported mild symptoms of contact dermatitis (itchy skin or irritation) on the location of the IMU. Future work will include screening for history of allergy, skin reaction to adhesive, or skin condition that could be triggered by contact with adhesive (e.g., eczema) as exclusion criteria.

### Recommendations for improvement

This study was part of a larger project aiming to model motor symptoms and mobility in response to medication intake in PwP and has provided vital insights for the future. Firstly, technical issues (notifications received late, not received, or received more than once) with the smartwatch need to be addressed. To this aim, we will update the smartphone to the latest version of the android mobile operating system and upgrade the smartwatch to the most recent model, which may improve the timing of notification delivery. We could not identify why many expected interactions were missing (*n* = 90), or received multiple times (*n* = 191) with the current system. These might be due to a system failure, with either no notification or multiple ones being sent to, or received by, the smartwatch. The Aeqora application will be updated which should improve notification delivery. Alternatively, these missing or repeated interactions might be due to participants not acknowledging their medication intake or inputting the same intake several times. With the current system there were only two possible outcomes for medication intake, either “Yes” when participants acknowledged medication intake or “No” when participants acknowledged not taking their medication. We will add a “No interaction” outcome so that in the future we can distinguish whether participants received the notification but ignored it (“No interaction”) or if the notification had not been sent to or received by the smartwatch (system error). In addition, participants may have received their notifications but not felt the watch vibration, possibly due to a higher sensory threshold (45). To minimise this potential risk of losing data, in the future, we will trial different notification types with participants and let them choose which pattern they better detect (vibration only, auditory alarm only, vibration and auditory alarm). Finally, we will add a “thank you” message confirming to participants that their input has been recorded which should prevent repeated inputs.

### Limitations

The present study utilised an indirect approach to assess medication intake which relies on participants self-reporting their intakes and may be considered less accurate than direct observation of intakes, or invasive and potentially expensive laboratory detection of the active substance (15). This indirect method was chosen because it is easily applicable to large cohorts, but it requires reliable interactions with the smartwatch which may be difficult to achieve for individuals with advanced PD and may be susceptible to active deception (i.e., participant choosing not to consume the medication whilst acknowledging its intake) (15). In the future to reduce this risk, our system could be associated with medication specific upper arm movement detection algorithms, such as those developed by other authors (51–53).

Improvements made to the smartwatch should improve both the feasibility and usability aspect of the DHTS but further work is needed to quantify the progress made. In the future, additional practice time will be scheduled to ensure participants have sufficient understanding of how to use the devices, and for technical issues to be identified and resolved.

This study presents data collected from a relatively small sample, which only included participants at stage I (*n* = 1) and II (*n* = 29) of the Hoehn and Yahr Scale. The majority of participants (*n* = 16) were classified as not frail (only four participants had two or more frailty characteristics), with little OFF-time (median time = 2 h) or dyskinesia periods (median time = 0 h) and did not have severe cognitive impairment (all participants scored ≥21/30 on the MoCA). Additionally, multimorbidity frequently coexists with PD (54), but this was not recorded in the present study. Furthermore, the sample was recruited from a regional movement disorder clinic with specialised PD expertise. Therefore, this study only reflects people in the early stages of the disease with mild to moderate motor and cognitive symptoms and findings cannot be generalised to the wider PD population. Hence, any attempt at utilising this DHTS with people in the later stage of the disease should be preceded by a feasibility study conducted with the intended population. Similarly, this DHTS may not be adapted to the study of people in the very early stages of the disease (prodromal and Hoehn and Yahr stage I) as it may be seen as too constraining and of limited relevance to them if prescribed less complex medication regimens.

## Conclusion

This study demonstrated that assessing self-reported medication adherence, tracking motor complications, and monitoring mobility in people with mild-to-moderate Parkinson's disease are feasible using this novel DHTS and a motor complications diary. Analysis of questionnaire answers and qualitative feedback highlighted contrasting opinions on the DHTS's usability. Specifically, the IMU and smartphone were considered usable by most participants, but difficulties arose when interacting with the smartwatch due to technical issues, lack of familiarity with the system and motor symptoms (tremor). In the future, the DHTS will be improved to allow for more reliable monitoring of medication intakes, which should enhance our capacity to model motor symptoms, complications, and their fluctuation in response to medication intake. This will provide greater insights for clinicians to optimise complex medication regimens in individuals with PD, potentially improving their quality of life.

## Data availability statement

The datasets supporting the conclusions of this article can be made available by the corresponding author upon reasonable request.

## Ethics statement

The studies involving human participants were reviewed and approved by London—Westminster Research Ethics Committee (REC reference: 21/PR/0469). The patients/participants provided their written informed consent to participate in this study.

## Author contributions

Study design: SDD, LA, AY, and LR. Data collection and pre-processing of the data: HB, PB, HH, and LA. Ethical approval, people with Parkinson's recruitment, and clinical oversight: SDD, HB, PB, HH, LA, AY, and LR. Data analysis, statistical analyses, and tables creation: HD, EP, and EB. Figures preparation and drafting of the manuscript: HD and EP. Data interpretation: HD, EP, LA, and SDD. Intellectual contribution: HD, EP, EB, HB, RMA, RD, JE, MN, FC, NI, JS, AY, LR, LA, and SDD. All authors have provided critical intellectual input during the revision of the manuscript, have reviewed the manuscript, and approved the submitted version.
