# Supplementary material for: Feasibility and usability of a digital health technology system to monitor mobility and assess medication adherence in mild-to-moderate Parkinson's disease
Source: Front Neurol. 2023 Mar 15;14:1111260. doi: 10.3389/fneur.2023.1111260 (PMC10050691; doi:10.3389/fneur.2023.1111260)
Supplement: Supplementary file 1 [file Data_Sheet_1.PDF]

CiC – Effect of medication on mobility in people with PD

Version 1.1; 14 May 2021; IRAS ID: 295771

Subject Initial |__||__| (F, S)

Subject ID |__|__|__|__|__|

**Day/ Time**	**6:00 7:00**		**7:00 8:00**		**8:00 9:00**		**9:00 10:00**		**10:00 11:00**		**11:00 12:00**		**12:00 13:00**		**13:00 14:00**		**14:00 15:00**		**15:00 16:00**		**16:00 17:00**		**17:00 18:00**		**18:00 19:00**		**19:00 20:00**		**20:00 21:00**		**21:00 22:00**		Comments
Monday																																	
Tuesday																																	
Wednesday																																	
Thursday																																	
Friday																																	
Saturday																																	
Sunday																																	

*Split cells indicate the “half an hour” – e.g. 6.30

Example: Off status (“O”) between 6.30 and 7.00 and dyskinesia (“D”) between 7.00 and 7.30 on Monday

**Day/ Time**	**6:00 7:00**		**7:00 8:00**		**8:00 9:00**	
Monday		O	D			
