# Supplementary material for: Feasibility and usability of a digital health technology system to monitor mobility and assess medication adherence in mild-to-moderate Parkinson's disease
Source: Front Neurol. 2023 Mar 15;14:1111260. doi: 10.3389/fneur.2023.1111260 (PMC10050691; doi:10.3389/fneur.2023.1111260)
Supplement: Supplementary file 2 [file Data_Sheet_2.PDF]

Usability questionnaire

Subject Initials |__||__|
F S

Subject ID |__|__|__|__|__|

English versions of questionnaires

Rabinovich

Please select a response for each of these questions that best reflects your experiences and opinions of the Wearable Technology System (Wearable device, smartwatch and smartphone).

Section A

<u>How much trouble did you have getting started with the Wearable Technology System?</u>

<u>(NOTE: 'getting started with' refers to the first few hours of wear/use in the week. It is how the participant felt while using/wearing it initially at the start of the week)</u>

O No trouble to start up

O Sometimes trouble to start up

O Regularly caused trouble to start up

O Always trouble to start up

O I had to call the centre to get help in starting-up

<u>The Wearable Technology System was easy to put on/take off</u>

O Yes this was very easy

O This worked just fine

O I found it somewhat difficult

O I found it difficult

O I was unable to manage this on my own

<u>I experienced technical problems with the Wearable Technology System</u>

O All the time

O Frequently

O Sometimes

O Seldom

O Never

Subject Initials |__||__|
F S

Subject ID |__|__|__|__|__|__|

The Wearable Technology System interfered with my normal activities

O All the time

O Frequently

O Sometimes

O Occasionally

O Never

I felt comfortable wearing the Wearable Technology System

O All the time

O Frequently

O Sometimes

O Occasionally

O Never

I felt embarrassed wearing the Wearable Technology System

O All the time

O Frequently

O Sometimes

O Occasionally

O Never

The instructions on how to use the Wearable Technology System were clear

O Strongly disagree

O Disagree

O Neutral

O Agree

O Strongly agree

Subject Initials |__||__|
F S

Subject ID |__|__|__|__|__|

Using the Wearable Technology System on a daily basis was easy

O Strongly disagree

O Disagree

O Neutral

O Agree

O Strongly agree

The Wearable Technology System was bulky/heavy.

O Yes , very much so

O Yes much

O Not particularly

O Not at all

O No opinion

The Wearable Technology System (Wearable device) bothered me in the bed.

O Yes , very much so

O Yes much

O Not particularly

O Not at all

O No opinion, I did not wear the monitor at night

I felt my privacy was invaded by the Wearable Technology System

O Strongly disagree

O Disagree

O Neutral

O Agree

O Strongly agree

Subject Initials |__||__|
F S

Subject ID |__|__|__|__|__|

<u>If my doctor would like to use the Wearable Technology System to assess my activity and medication adherence I would be willing to wear it and use it for</u>

O Less than 1 day

O 2-4 days

O 1week

O Longer than 1 week.

O I would not mind wearing the monitor continuously (longer than 1 month)

Section B

We want to ask you to provide a final score for the Wearable Technology System.

All things considered can you give the device a score from 0% to 100%, where 0 means the worst possible system and 100% means the ideal system in your opinion.

<u>My score for Wearable Technology System</u>: .... /100

In the section below we want to give you the opportunity to give other comments on the Wearable Technology System and its component (wearable device (lower back), smartwatch and smartphone).

Wearable device:

Smartwatch:

Smartphone:

WT as a whole:

I experienced the following problems with the Wearable Technology System:

Wearable device:

Smartwatch:

Smartphone:

WT as a whole:

I liked these features of the Wearable Technology System in particular:

Wearable device:

Smartwatch:

Smartphone:

WT as a whole:
